# Sinomenine Protects against Lipopolysaccharide-Induced Acute Lung Injury in Mice via Adenosine A_2A_ Receptor Signaling

**DOI:** 10.1371/journal.pone.0059257

**Published:** 2013-03-15

**Authors:** Jun Li, Li Zhao, Xie He, Yi-Jun Zeng, Shuang-Shuang Dai

**Affiliations:** 1 Department of Cardiothoracic Surgery, Southwest Hospital, Third Military Medical University, Chongqing, China; 2 Department of Biochemistry and Molecular Biology, Third Military Medical University, Chongqing, China; Vanderbilt University Medical Center, United States of America

## Abstract

Sinomenine (SIN) is a bioactive alkaloid extracted from the Chinese medicinal plant *Sinomenium acutum*, which is widely used in the clinical treatment of rheumatoid arthritis (RA). However, its role in acute lung injury (ALI) is unclear. In this study, we investigate the role of SIN in lipopolysaccharide (LPS)-induced ALI in mice. After ALI, lung water content and histological signs of pulmonary injury were attenuated, whereas the PaO_2_/FIO_2_ (P/F) ratios were elevated significantly in the mice pretreated with SIN. Additionally, SIN markedly inhibited inflammatory cytokine TNF-α and IL-1β expression levels as well as neutrophil infiltration in the lung tissues of the mice. Microarray analysis and real-time PCR showed that SIN treatment upregulated adenosine A_2A_ receptor (A_2A_R) expression, and the protective effect of SIN was abolished in A_2A_R knockout mice. Further investigation in isolated mouse neutrophils confirmed the upregulation of A_2A_R by SIN and showed that A_2A_R-cAMP-PKA signaling was involved in the anti-inflammatory effect of SIN. Taken together, these findings demonstrate an A_2A_R-associated anti-inflammatory effect and the protective role of SIN in ALI, which suggests a potential novel approach to treat ALI.

## Introduction

Acute lung injury (ALI) is a life-threatening condition that can develop during the course of several clinical situations such as pneumonia, major trauma, acid aspiration, and sepsis [1], [2]. The pathogenesis of ALI is characterized by an influx of protein-rich fluid into the interstitial and intra-alveolar spaces as a result of increased permeability of the alveolar-capillary barrier in conjunction with excessive invasion of inflammatory cells — particularly neutrophils [3]–[5]. Accordingly, the inflammatory response plays an essential role in the progression of ALI.

Sinomenine (7,8-didehydro-4-hydroxy-3,7-dimethoxy-17-methylmorphinane-6- one, SIN) is a biomonomer alkali derived from the Chinese medicinal plant *Sinomenium acutum*. Traditionally, SIN has been used in the treatment of rheumatoid arthritis due to its role in inhibition of leukocytes migration across blood vessel walls, and its histamine-releasing and anti-angiogenic properties [6]–[8]. Recently, the anti-inflammatory and immunorepressive effects of SIN have attracted more and more attention. Studies *in vitro* have shown that SIN is able to inhibit lymphocyte proliferation and antibody production by B cells and potently reduce the production of inflammatory factors by macrophages [9]–[11]. Using an animal model, Kondo *et al.* demonstrated that SIN protected mice from endotoxin-induced fulminant hepatitis by suppressing TNF production and/or reactive oxygen generation [12]. In addition, in a cardiac allograft transplantation model, SIN was found to dose-dependently attenuate thymidine incorporation, interleukin-2 synthesis, and cell cycle progression of activated T-lymphocytes, which prolonged cardiac allograft survival and block tissue remodeling of chronic cardiac allograft rejection [13]–[16]. These reports lead us to hypothesize that SIN may also play a protective role in ALI because of its anti-inflammatory effect.

Adenosine A_2A_ receptor (A_2A_R) is one of the four well-known adenosine receptors (A_1_, A_2A_, A_2B_ and A_3_), which belongs to the family of the G-protein-coupled receptor. Recent studies have demonstrated that A_2A_R is widely expressed in the lung and play a protective role during ALI [17], [18]. The anti-inflammatory effect is confirmed to account for this A_2A_R-mediated protection in several ALI models, such as LPS-induced lung injury [19], or in models of lung injury induced by pulmonary ischemia reperfusion injury [20] or lung transplantation [21]. Attenuation of the inflammatory response and facilitation of subsequent repair by A_2A_R in the lung can be targeted to numerous sites, which include neutrophils, resident macrophages, bronchial epithelial cells, mast cells and lymphocytes [22]–[26]. Since most of these responsive cells are also reported to be regulated by SIN as described above and both SIN and A_2A_R are anti-inflammatory, it prompts us to investigate whether regulation of A_2A_R is involved in the SIN effect in ALI.

Accordingly, in this study, to elucidate the role of SIN in ALI and the possible link between SIN and A_2A_R in ALI, we constructed a LPS-induced ALI model in both wild type (WT) and A_2A_R gene knockout (KO) mice, and investigated the effect of SIN on lung water content, the PaO_2_/FIO_2_ (P/F) ratio, histological signs of pulmonary injury, neutrophil infiltration and expression of the inflammatory cytokines TNF-α and IL-1β. Furthermore, being the critical responsive cell type in ALI, neutrophils were isolated from WT and A_2A_R KO mice to investigate the associated mechanism for the effect of SIN on ALI.

## Materials and Methods

### Animals

Global A_2A_R homozygous knockout (KO) mice and their WT littermates were obtained from Dr. Jiang-Fan Chen (Boston University School of Medicine) and were generated as previously described [27]–[29]. Before the experiments, mice were housed under 12 h light/dark conditions with free access to food and water in the Experimental Center of Medical Animals of the Daping Hospital/Research Institute of Surgery, the Third Military Medical University (Chongqing, China). All procedures used in this study were approved by the Institutional Animal Care and Use Committee of the Third Military Medical University.

### Induction of acute lung injury and drug administration

Lipopolysaccharide (LPS) was purchased from Sigma (St. Louis, MO), and SIN was purchased from Xisenfo Biotechnology Company (Shanxi, China). Experimental mice (8–10 weeks old) were anesthetized with 1.5% sodium pentobarbital followed by intratracheal administration of 50 µg LPS from *Escherichia coli* (serotype O111:B4; Sigma-Aldrich) in 40 µl PBS via a 20-gauge intravenous catheter [30]. Different doses of SIN (30, 60 and 120 mg/kg) were given to the mice by intraperitoneal injection (i.p.) 1 hour before LPS treatment. Mice treated intratracheally with the vehicle, 40 µl PBS, served as controls.

### Assay of lung water content

At 24 hour post-LPS injection, the lungs of the injured mice were harvested, and the lung water content was assayed. The trachea and esophagus were removed by blunt dissection, and the wet weight of the lungs was determined. Subsequently, the lungs were incubated at 55°C overnight to remove all moisture. The dry weight was then measured, and the percentage of water content in lung was calculated by the formula (wet weight-dry weight)/wet weight×100%.

### Blood gas analysis

To assess the pulmonary gas exchange, blood gas analyses were performed in subsets of experiments by obtaining arterial blood. A lateral thoracotomy was performed to access the left ventricle, and the blood was obtained via cardiac puncture. The analysis was performed immediately after collection with an I-STAT Analyzer (Abbott Point, Ottawa, Ontario, Canada), and the arterial partial pressure of oxygen was measured.

### Histopathological evaluation

Mice were anesthetized at 24 hours after injury and killed transcardially with saline, followed by treatment with 4% paraformaldehyde. Lungs were immediately removed and post-fixed in 4% paraformaldehyde for 24 hours. Paraffin-embedded sections (5 µm thick) were stained with hematoxylin and eosin (HE) for visualization under a light microscope at 200× magnification.

### Immunofluorescence

At 24 hour post-injury, neutrophil infiltration in lung tissue of mice was determined with standard immunofluorescence immunohistochemistry and analyzed as described previously [30]. Briefly, the frozen sections of injured lung tissue were fixed in acetone for 10 min, followed by incubation with rabbit anti-mouse CD177 antibody (Santa Cruz, CA, USA, 1:500), which labels neutrophils, on individual slides for 30 min at 37°C. After three washes with PBS and incubation with FITC-conjugated goat anti-rabbit secondary antibody for 30 min at 37°C, the sections were washed again in a similar manner and examined by fluorescence microscopy at 200× magnification. Cells were considered to be positive for CD177 if specific fluorescence was observed. Nuclei were after stained by 4'-6-diamidino-2-phenylindole (DAPI) to show total cells. The results were analyzed by Image-Pro plus version 6.0 and index of CD177 positive cells was presented to indicate the amount of infiltrated neutrophils.

### ELISA

At 24 hour after injury, quantification of the protein levels of TNF-α and IL-1β in the lung tissue was performed with commercially available ELISA kits (Boster, Wuhan, China) in accordance with the manufacturer's instructions.

### Microarray

Total RNA from each sample was quantified by the NanoDrop ND-1000, and RNA integrity was assessed by standard denaturing agarose gel electrophoresis. For microarray analysis, the Agilent Array platform was employed. The sample preparation and microarray hybridization were performed based on the manufacturer's standard protocols with minor modifications. Briefly, mRNA was purified from 1 µg total RNA after removal of rRNA (mRNA-ONLY™ Eukaryotic mRNA Isolation Kit, Epicentre). Each sample was then amplified and transcribed into fluorescent cRNA along the entire length of the transcripts without 3’ bias utilizing a random priming method. The labeled cRNAs were hybridized onto the Human LncRNA Array v2.0 (8×60K, Arraystar), which represented all long transcripts, both protein coding mRNAs and lncRNAs. After washing the slides, the arrays were scanned by the Agilent Scanner G2505B. Agilent Feature Extraction software (version 10.7.3.1) was used to analyze acquired array images. Quantile normalization and subsequent data processing were performed using the GeneSpring GX v11.5.1 software package (Agilent Technologies). Differentially expressed mRNAs were identified through fold-change filtering. Pathway analysis and gene ontology (GO) analysis were applied to determine the roles of these differentially expressed mRNAs in either these biological pathways or GO terms. Finally, hierarchical clustering was performed to show the distinguishable mRNA expression patterns among the samples.

### Isolation of neutrophils and treatment

Mouse neutrophils were isolated from the bone marrow of 6- to 8-week-old wild-type and A_2A_R KO mice using a discontinuous Percoll gradient according to standard procedures [31]. Plasma and mononuclear cells were removed from the buffy coat by aspiration after centrifugation (400×g for 20 min) at 25°C. Erythrocytes were removed using a 2% gelatin sedimentation technique. Residual erythrocytes were removed by lysis in cold NH_4_Cl buffer. The remaining cells were > 90% neutrophils as assessed by microscopic evaluation. Neutrophils were stimulated with LPS (100 ng/ml) [32], and SIN (1 mol/L) [33] was added 30 minutes before LPS treatment. To detect the downstream signaling, 10 µmol/L of N-[2-[[3-(4-bromophenyl)-2-prope-nyl]amino]ethyl]-5-isoquinolinesulfonamide dihydrate dihydrochloride (H-89, Calbiochem, Germany) [34], an inhibitor of protein kinase A (PKA), was used. Neutrophils with only PBS treatment were served as control. All of the studies with the neutrophils were performed within 6 h of isolation.

### Real-time PCR

Total RNA of either lung tissue or neutrophils was isolated using TRIzol (Invitrogen, Carlsbad, CA, USA) according to the manufacturer's guidelines and reverse transcribed. Quantitative PCR amplification was carried out in triplicate using a SYBR Green kit (TaKaRa Bio Inc., Dalian, China). Primers sets (shown as sense, antisense) for the following genes were A_1_R (5′-GTGTCCTTCTGCGTCCTGGTATG-3′, 5′-CTTGCTCTCCCTTCCTCGTTTGG-3′); A_2A_R (5′-AGCCAGGGGTTACATCTGTG-3′, 5′-TACAGACAGCCTCGACATGTG-3′); A_2B_R (5′-GGAAGGACTTCGTCTCTCCA-3′, 5′-GGGCAGCAACTCAGAAAACT-3′); A3R (5′-CAATTCGCTCCTTCTGTTCC-3′, 5′-TCCCTGATTACCACGGACTC-3′; TNF-α (5′-CTGTGAAGGGAATGGGTGTT-3′, 5′-TCACTGTCCCAGCATCTTGT-3′); and IL-1 (5′-ACTGTTTCTAATGCCTTCCC-3′, 5′-ATGGTTTCTTGTGACCCTGA-3′). An endogenous control gene, GAPDH, was included in each quantitative PCR analysis. The relative abundance of the target genes was obtained by comparison to a standard curve generated by a serial dilution of reference cDNA from a normal mouse and normalized to GAPDH.

### Detection of cAMP levels

The cAMP levels in the LPS-stimulated neutrophils were determined at 4 h post-LPS treatment using the overnight acetylation protocol of a [^125^I]-cAMP SPA kit (Amersham Bioscience, Sweden). This protocol is based on a cAMP competitive binding assay.

### Statistical analyses

Graphic data are expressed as the mean±S.E.M. Statistical analysis of the data was performed by one-way ANOVA followed by Bonferroni post hoc test. Significance levels were set at *P*<0.05 for all statistical analyses.

## Results

### Sinomenine attenuates lung damage in mice after ALI

**Figure 1:**
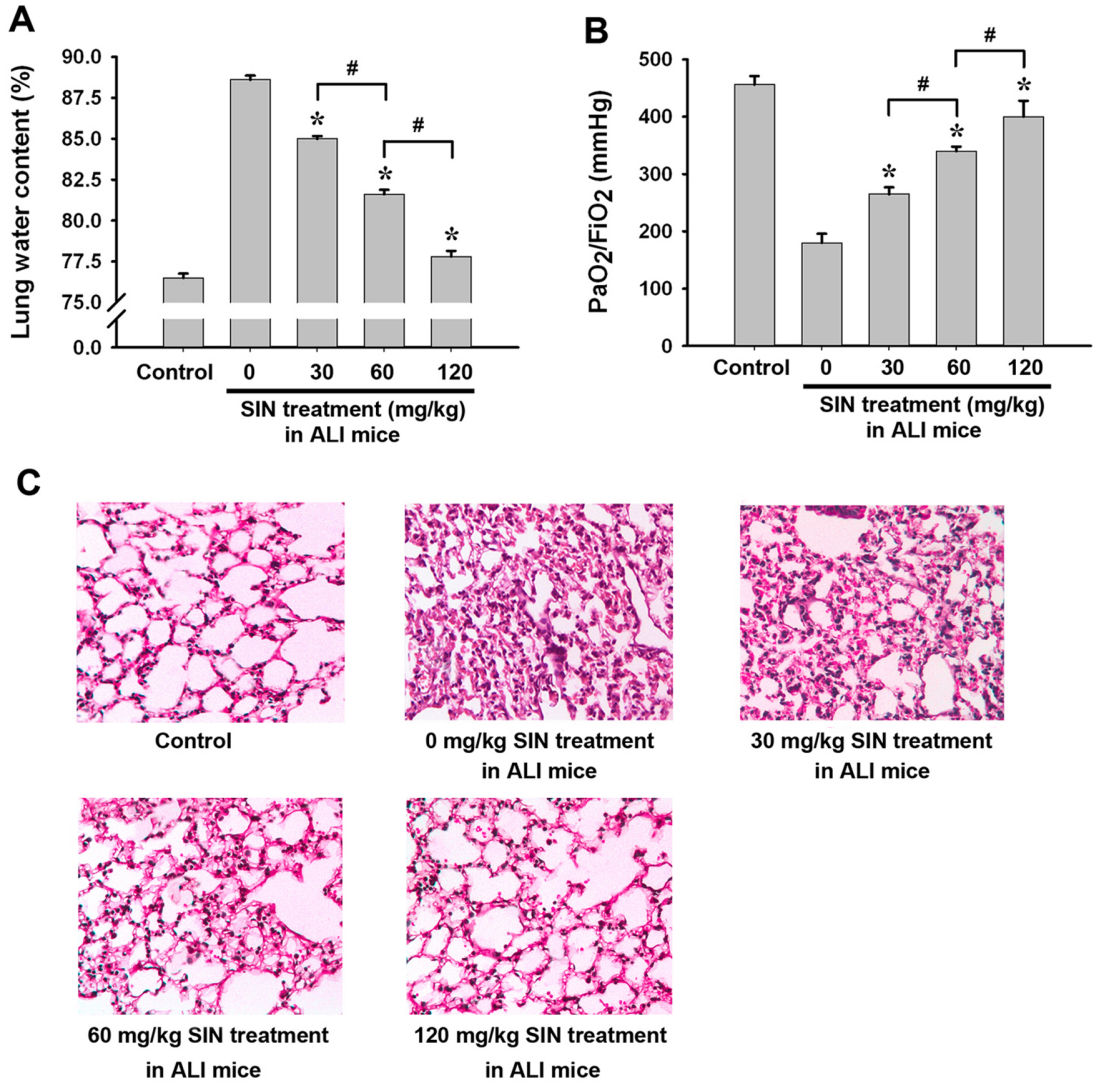
SIN attenuates lung damage at 24 hour after acute lung injury in a dose-dependent manner. One hour before LPS injection, 0, 30, 60 or 120 mg/kg SIN was administrated to mice. At 24 hour after ALI, lung damage was assessed. The mice intratracheally treated with 40 µl PBS were served as the control. (A) Lung water content. (B) PaO_2_/FIO_2_ (P/F) ratio. (C) HE staining for histopathological changes in lung tissues. * *p*<0.01 compared to the injured group without SIN treatment (0 mg/kg SIN treatment); # *p*<0.01 compared to the two indicated groups; NS: no significant difference between the two groups. (n = 8∼10 mice per group).

Twenty-four hours after ALI, lung damage in the mice was assessed. The data show that SIN (30, 60 and 120 mg/kg) reduced lung water content (Figure 1A) and elevated PaO_2_/FIO_2_ (P/F) ratios (Figure 1B) in a dose-dependent manner. The HE staining of murine lung tissue sections showed LPS treatment induced severe lung damages including disfiguration of alveoli, infiltration of multiple cells, pulmonary exudation and consolidation of lung tissues. However, 30 mg/kg of SIN treatment mildly, 60 mg/kg of SIN treatment moderately, and 120 mg/kg of SIN treatment significantly attenuated these histological signs of pulmonary injury (Figure 1C), respectively. These data suggest that SIN protects against LPS-induced lung damage in a dose-dependent manner.

### Neutrophil infiltration is reduced by sinomenine treatment

**Figure 2:**
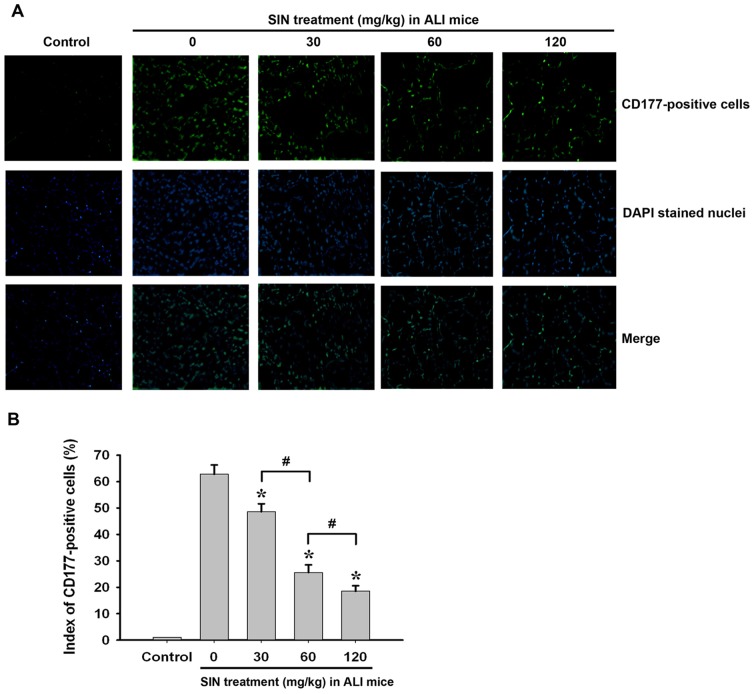
SIN inhibits neutrophil infiltration at 24 hour after acute lung injury in a dose-dependent manner. Neutrophil infiltration into lung tissue was evaluated at 24 hour after acute lung injury by immunofluorescence using a CD177 primary antibody and a FITC-conjugated secondary antibody. Mice treated intratracheally with 40 µl PBS served as controls. (A) CD177-positive cells in the lung of control injured mice. (B) Cell counting and statistical analysis. * *p*<0.01 compared to the injured group without SIN treatment (0 mg/kg SIN treatment); # *p*<0.01 compared to the two indicated groups; NS: no significant difference between the two groups. (n = 8∼10 mice per group).

At 24 hour post-ALI, neutrophil infiltration into the lung tissues was detected with the neutrophil-specific marker CD177. As shown in Figure 2, multiple CD177-positive cells were observed in the lung tissue of mice with LPS-induced ALI when compared with the control group. However, 30 mg/kg SIN treatment significantly reduced the CD177-positive cells, which indicated that neutrophil recruitment into lung tissue of mice was suppressed to some extent. This inhibition of neutrophil infiltration was found to be more obvious in the lung sections of mice after administration of either 60 or 120 mg/kg SIN. These results indicate that SIN plays a significant anti-inflammatory role in LPS-induced ALI.

### Sinomenine inhibits inflammatory cytokine expression

**Figure 3:**
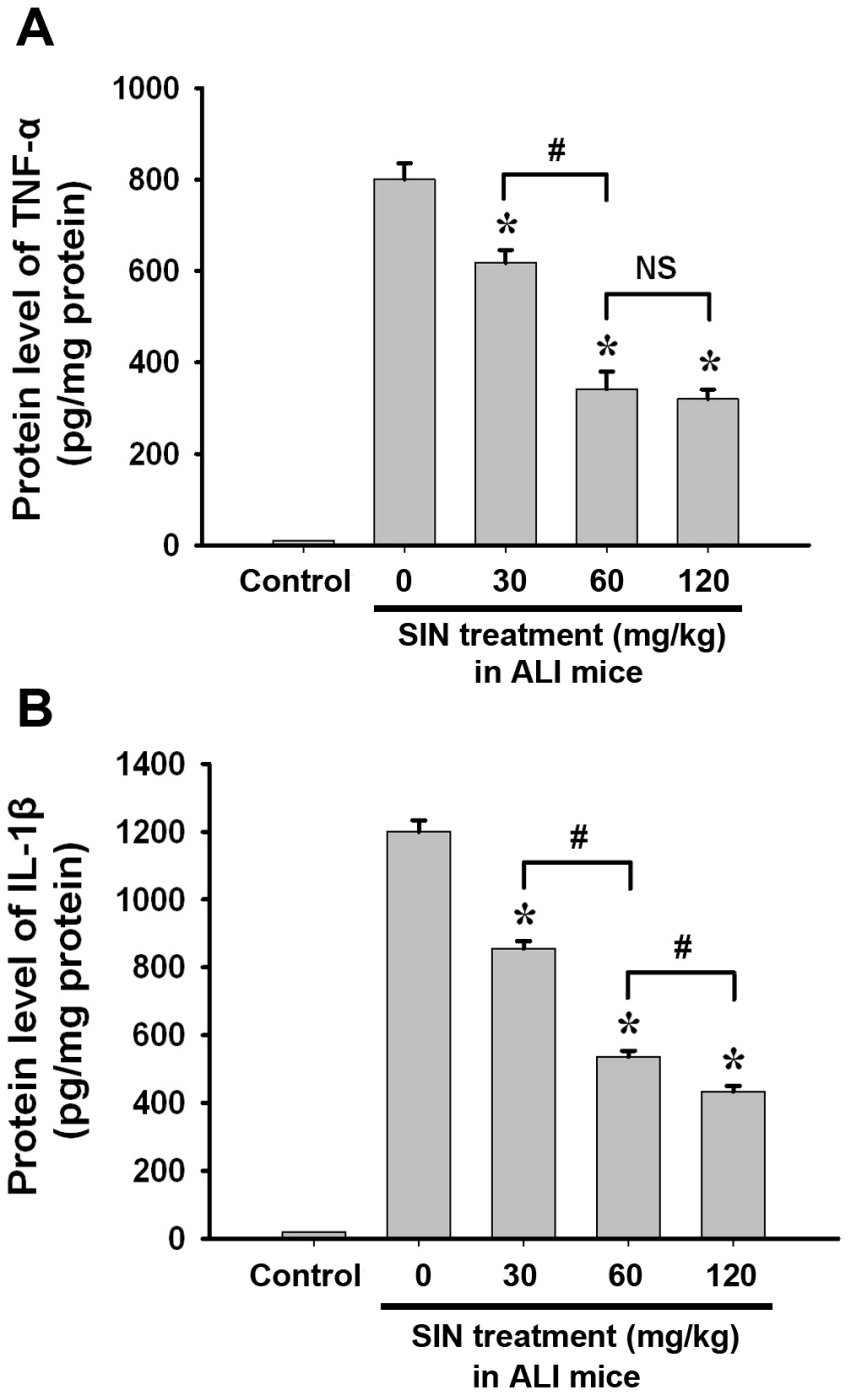
SIN suppresses acute lung injury-induced TNF-α and IL-1β expression levels in a dose-dependent manner. The protein expression levels of TNF-α and IL-1β in injured murine lung tissue were assayed at 24 hour post-acute lung injury by ELISA. Mice treated intratracheally with 40 µl PBS served as controls. (A) TNF-α protein levels. (B) IL-1β protein levels. * *p*<0.01 compared to the injured group without SIN treatment (0 mg/kg SIN treatment); # *p*<0.01 compared to the two indicated groups; NS: no significant difference between the two groups. (n = 8∼10 mice per group).

Inflammation is the critical pathological course in the progression of ALI. To further confirm the anti-inflammatory effect of SIN, we assayed the protein expression of the inflammatory cytokines TNF-α and IL-1β in murine lung tissues. At 24 hours post-injury, SIN treatment significantly reduced both TNF-α (Figure 3A) and IL-1β protein levels (Figure 3B) in the mice suffered from LPS-induced ALI. This effect of SIN was observed in a dose-dependent manner (Figure 3). These results indicate that the anti-inflammatory effect of SIN may be responsible for its protection against ALI.

### Sinomenine upregulates adenosine A_2A_R expression in lung tissues of mice

**Figure 4:**
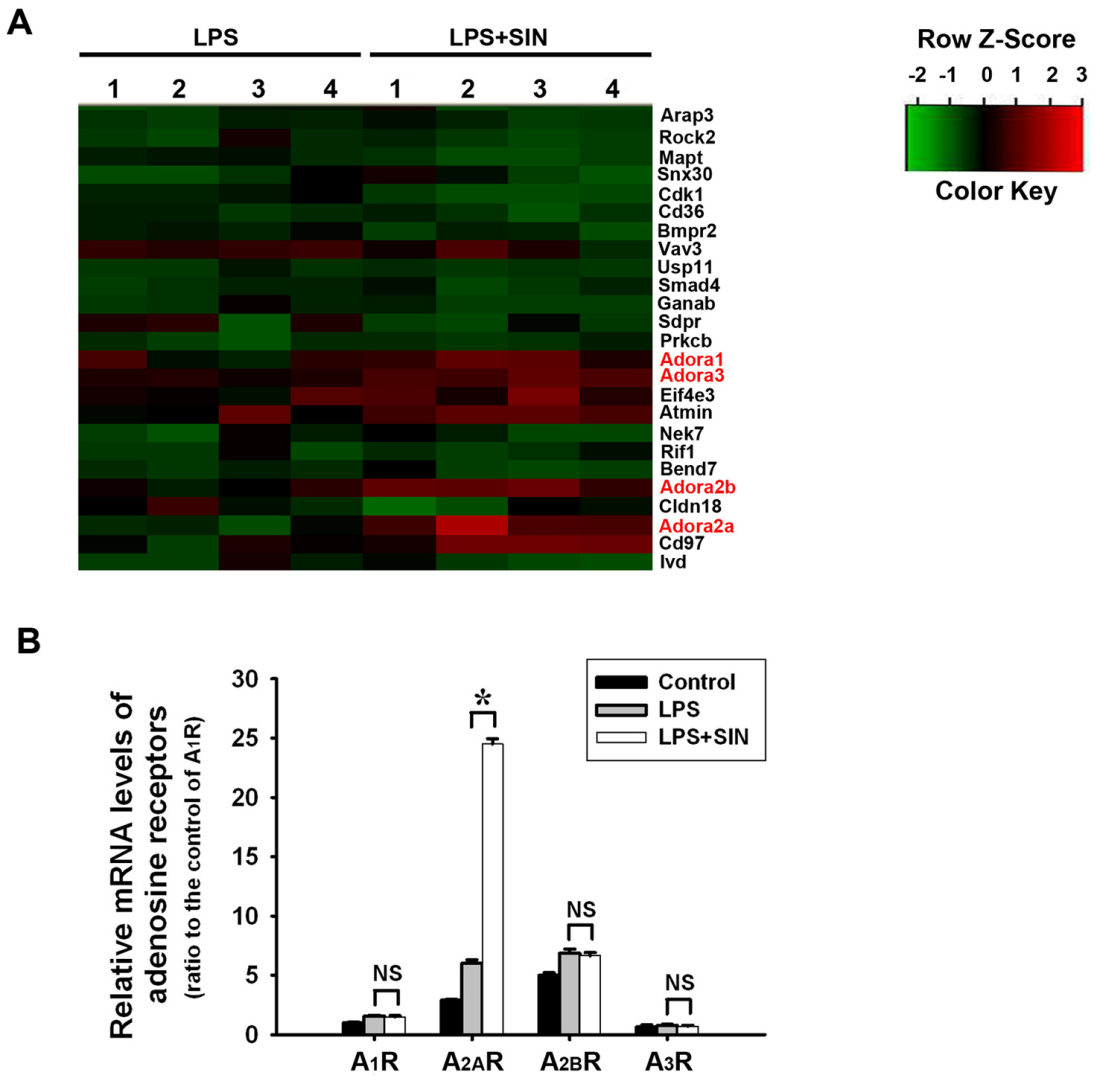
A_2A_R expression in lung tissues is upregulated by SIN. Expression profiles of murine lung tissues with or without SIN treatment were assayed by microarray and validated by real-time PCR. (A) Expression profiles in the murine lung tissue. The selected genes showed different expression patterns for the mice either with or without SIN treatment. LPS: LPS-induced ALI mice without SIN treatment; LPS+SIN: LPS-induced ALI mice pre-treated with 120 mg/kg SIN (n = 4 mice per group for microarray). (B) Relative A_1_R, A_2A_R, A_2B_R and A_3_R mRNA expression levels. Mice treated intratracheally with 40 µl PBS served as controls. * *p*<0.01 compared to LPS-induced ALI group without SIN treatment; # *p*<0.01 compared to the two indicated groups; NS: no significant difference between the two groups. (n = 8∼10 mice per group).

To investigate the possible mechanism of SIN's role in ALI, we detected gene expression changes in the lung tissues of ALI mice without SIN treatment and ALI mice with 120 mg/kg SIN. The microarray results indicated that the expression of four adenosine receptors (A_1_, A_2A_, A_2B_ and A_3_ receptors) were significantly increased in the tissue that received 120 mg/kg SIN treatment compared to the control group (Figure 4A). To validate this result, real-time PCR was performed to assay the mRNA levels of the four adenosine receptors in murine lung tissues. Only A_2A_R mRNA expression was markedly elevated by SIN treatment, whereas the A_1_ receptor (A_1_R), A_2B_ receptor (A_2B_R) and A_3_ receptor (A_3_R) were not (Figure 4B). These data suggest the upregulation of A_2A_R by SIN in ALI.

### Protective effects of sinomenine are largely blocked in A_2A_R knockout mice

**Figure 5:**
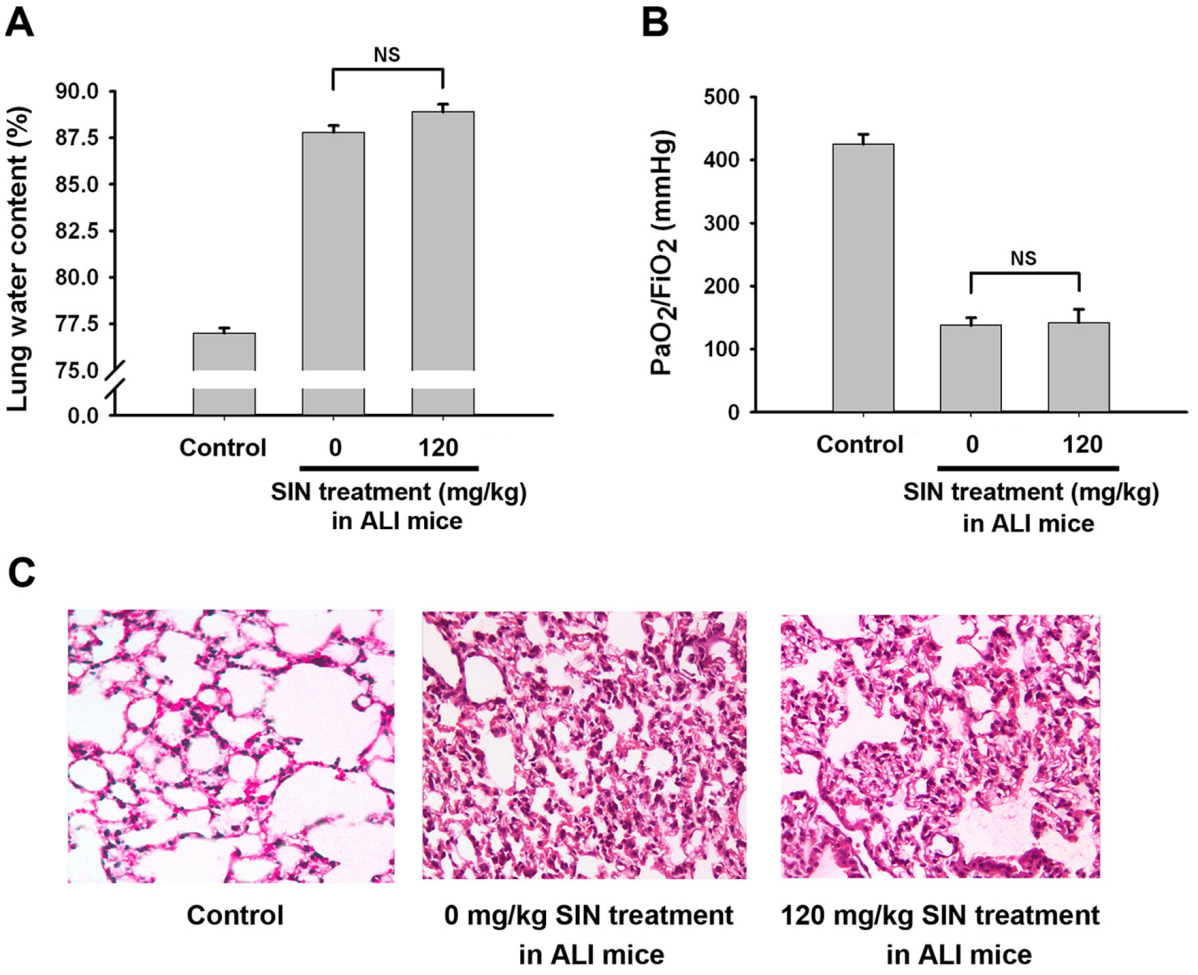
Protection of SIN against lung damages is not observed in A_2A_R KO mice. One hour before LPS injection to induce acute lung injury, 120 mg/kg SIN was administered to A_2A_R KO mice. At 24 hour after ALI, lung damage of both the injured control and SIN-treated A_2A_R KO mice were assayed. Normal A_2A_R KO mice treated intratracheally with 40 µl PBS served as controls. (A) Lung water content. (B) PaO_2_/FIO_2_ (P/F) ratio. (C) HE staining for the histopathological changes in the lung tissue. NS: no significant difference between the two groups. (n = 8∼10 mice per group).

The upregulation of A_2A_R by SIN prompts the investigation that SIN exerts its effect in ALI in an A_2A_R-dependent manner. When observing the effect of SIN (120 mg/kg) on lung damage in A_2A_R KO mice suffering from LPS-induced ALI, there was no significant difference in the lung water content (Figure 5A), P/F ratio (Figure 5B) and histological signs of pulmonary injury (Figure 5C) between non-SIN-treated group and the SIN-treated group. The protection of SIN was not observed in A_2A_R KO mice, which indicates that A_2A_R is involved in the SIN protective role in ALI.

### Anti-inflammatory effects of sinomenine are abolished in A_2A_R knockout mice

**Figure 6:**
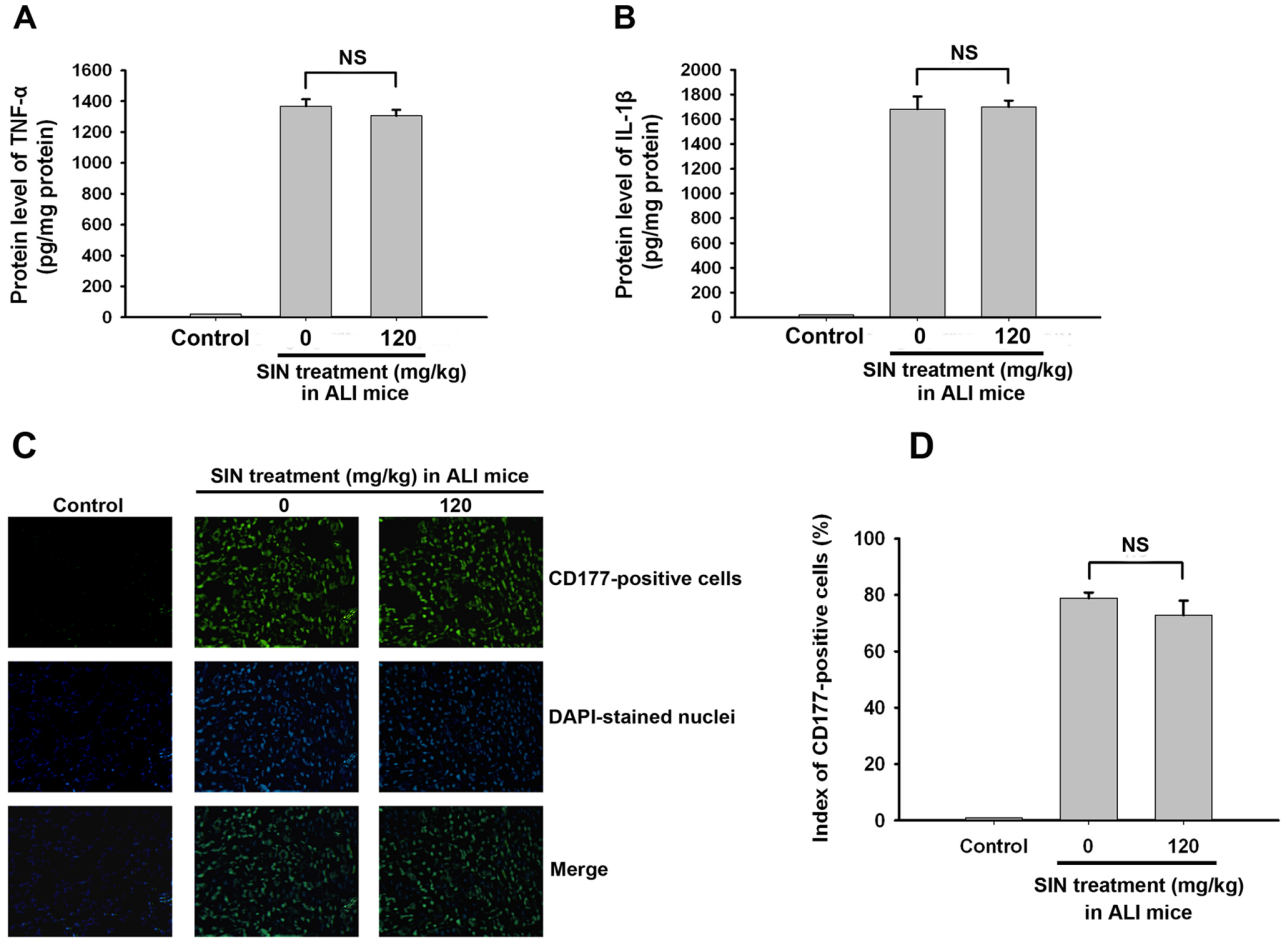
The anti-inflammatory effect of SIN is abolished in A_2A_R KO mice. Thirty minutes before LPS injection to induce acute lung injury, 120 mg/kg SIN was administered to A_2A_R KO mice. At 24 hour after ALI, neutrophil infiltration was detected by immunofluorescence using a CD177 primary antibody and a FITC-conjugated secondary antibody, and the protein expression levels of the inflammatory cytokines TNF-α and IL-1β were assayed by ELISA. The normal A_2A_R KO mice intratracheally treated with 40 µl PBS were served as the control. (A) TNF-α protein levels. (B) IL-1β protein levels. (C) CD177-positive cells in the murine lung tissue. (D) Cell counting and statistical analysis. NS: no significant difference between the two groups. (n = 8∼10 mice per group).

The effect of SIN on neutrophil infiltration and the expression levels of the inflammatory cytokines TNF-α and IL-1β were also assayed in A_2A_R KO mice at 24 hours post-injury. The suppression of TNF-α and IL-1β mRNA expression levels (Figure 6A and 6B) and the inhibition of neutrophil infiltration (Figure 6C and 6D) were not observed in the injured A_2A_R KO mice treated with SIN compared to the injured A_2A_R KO without SIN administration. These results confirm that the anti-inflammatory effect of SIN is largely mediated by A_2A_R.

### Sinomenine suppresses LPS-induced inflammatory cytokine expression in neutrophils in an A_2A_R-dependent manner

**Figure 7:**
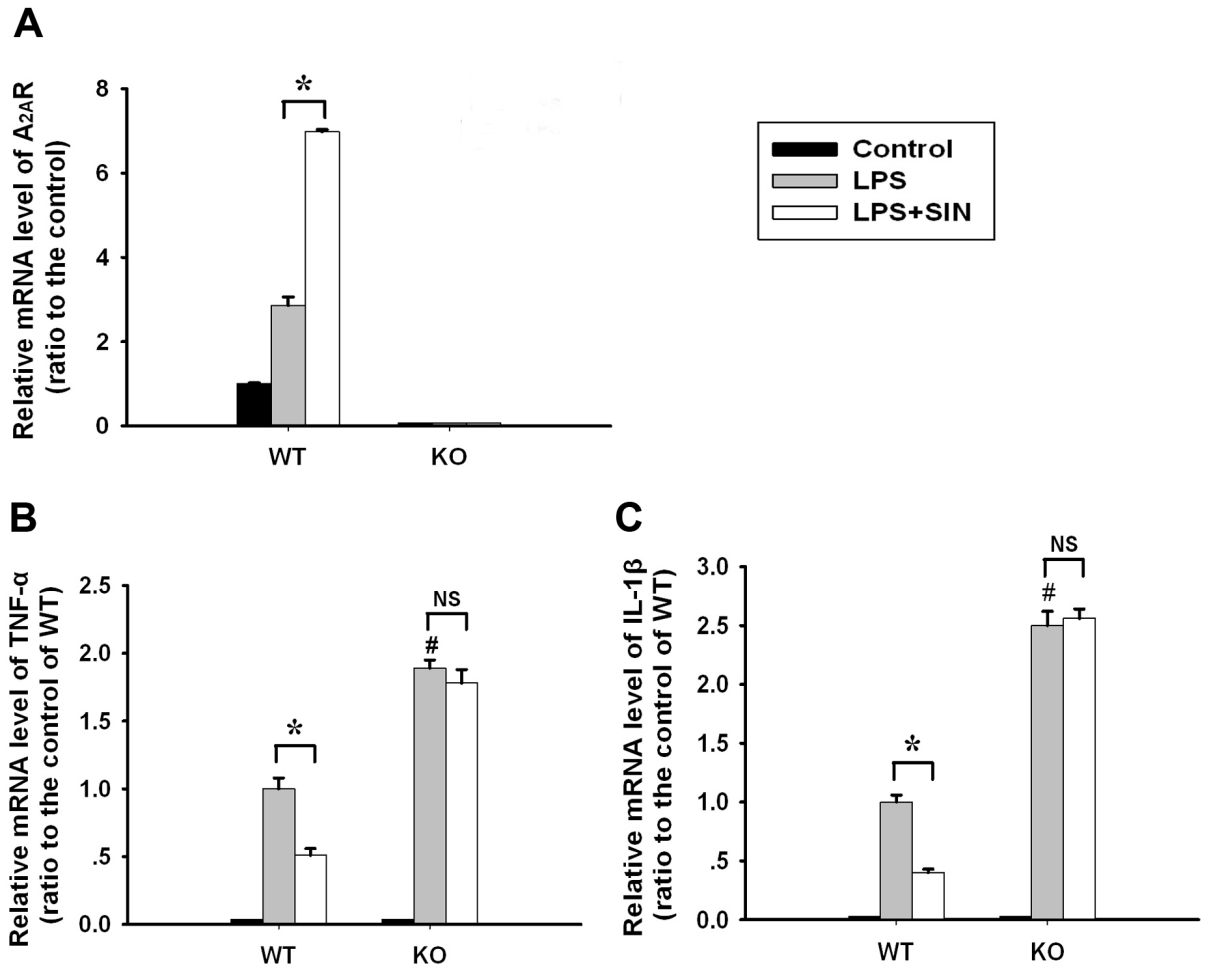
SIN inhibits inflammatory cytokine expression levels in LPS-stimulated murine neutrophils in an A_2A_R-dependent manner. In isolated WT and A_2A_R KO murine neutrophils, SIN (1 mol/L) was applied 30 minutes before LPS stimulation. The neutrophils treated with PBS served as control. At 4 h after LPS administration, the A_2A_R-associated effects of SIN were detected. (A) Relative A_2A_R mRNA levels. (B) Relative TNF-α mRNA levels. (C) Relative IL-1β mRNA levels. * *p*<0.01 compared to the two groups; # *p*<0.01 compared to the LPS-treated WT neutrophil group; NS: no significant difference between the two groups. (n = 8∼10 mice per group).

Because they are the key responsive cell in ALI, murine neutrophils were isolated and investigated. As observed in the *in vivo* results, SIN significantly upregulated A_2A_R mRNA expression (Figure 7A) and inhibited the LPS-induced TNF-α and IL-1β expressions (Figure 7B and 7C) in LPS-stimulated WT neutrophils. However, in LPS-stimulated A_2A_R KO neutrophils, there were no significant differences in TNF-α and IL-1β expression levels between the control and SIN-treated group (Figure 7A, 7B and 7C). These data suggest that SIN suppresses LPS-induced inflammatory cytokine expression in neutrophils in an A_2A_R-dependent manner.

### A_2A_R-cAMP-PKA signaling is associated with the anti-inflammatory effect of sinomenine

**Figure 8:**
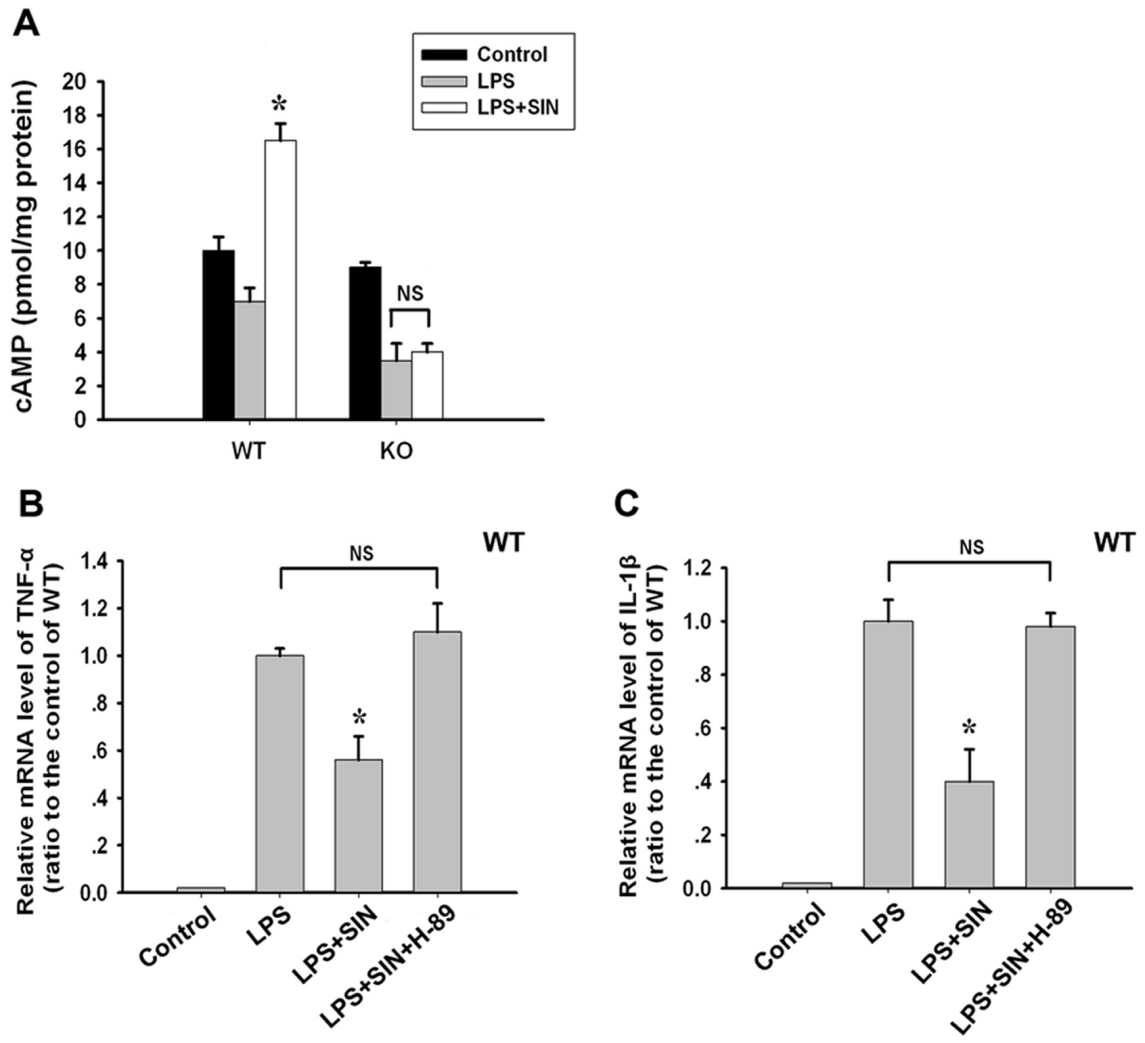
A_2A_R-cAMP-PKA signaling is involved in the anti-inflammatory effect of SIN. In isolated murine neutrophils, SIN (1 mol/L) was applied 30 minutes before LPS stimulation, and the A_2A_R-associated mechanism for the anti-inflammatory effect of SIN was further investigated by assaying for cAMP and using the PKA inhibitor H-89. The neutrophils treated with PBS were served as control. (A) cAMP levels. (B) TNF-α protein levels. (C) IL-1β protein levels. * *p*<0.01 compared to the LPS treatment groups; NS: no significant difference between the two groups. (n = 8∼10 mice per group).

In LPS-stimulated WT neutrophils, SIN was found to markedly increase cAMP levels, which was not observed in LPS-induced neutrophils from A_2A_R KO mice (Figure 8A). cAMP-PKA signaling is a major signal pathway of A_2A_R; thus, H-89, an inhibitor of PKA (cAMP downstream), was used in LPS-stimulated WT neutrophils. As expected, H-89 blocked the inhibitory effect of SIN on the expression of the LPS-induced inflammatory cytokines TNF-α and IL-1β (Figure 8B and 8C). These results indicate that the A_2A_R-associated cAMP-PKA signaling mediates the anti-inflammatory and protective role of SIN.

## Discussion

As a traditional Chinese medicine, purified SIN provides therapeutic efficacy and fewer side effects in patients with RA and mesangial proliferative nephritis, as confirmed from open clinical trials over the past 30 years [10], [35]. In the present study, we report for the first time that SIN significantly attenuates lung inflammatory damages in LPS-induced ALI. This protective effect of SIN is shown to be dependent on the upregulation of A_2A_R expression and the triggering of A_2A_R-associated cAMP-PKA signaling.

ALI is defined as an acute non-cardiogenic pulmonary edema. This is a result of an increase in the pulmonary microvascular permeability. Based on the degree of hypoxia, as indicated by the ratio of arterial oxygen partial pressure (PaO_2_) to the fraction of inspired oxygen (FiO_2_), ALI is defined by a PaO_2_/FiO_2_ gradient below 300 mmHg. In this study, intratracheal administration of LPS increased lung water content to approximately 87.5% (baseline 75%–78%) and decreased the P/F ratio to approximately 200 mmHg in mice. However, SIN treatment was found to significantly relieve LPS-induced lung edema, hypoxia and pathological changes in lung tissue in a dose-dependent manner. Previously, SIN has been shown to reduce the synthesis of prostaglandin E3, leukotriene C4, nitric oxide, and TNF-α by LPS-treated macrophages *in vitro* and in vivo [36]. SIN also decreases the expression of TNF-α and IL-1β in adjuvant-induced arthritic (AA) rats [37]. In this study, we for the first time demonstrate that in ALI, SIN efficiently suppresses the inflammatory response of neutrophil infiltration into the lung and inflammatory cytokine TNF-α and IL-1β expression levels in lung tissue, which confirmed the anti-inflammatory effect of SIN in tissue injuries and diseases.

Further investigation showed that SIN treatment in mice with ALI significantly upregulated A_2A_R expression. The A_2A_R is strongly expressed in the lung tissue and its anti-inflammatory protective role has been well-documented in lung injures [38]-[40]. For example, Sharma *et al.* found that in mouse lung ischemia-reperfusion (IR) injury model, the A_2A_R agonist ATL313 significantly attenuated the induction of TNF-alpha, KC (CXCL1), MIP-2 (CXCL2) and RANTES (CCL5) occurred after IR [20]. Thiel *et al.* reported that endogenous adenosine acts through A_2A_R reproducing the anti-inflammatory protective role in a model of LPS-induced acute respiratory distress syndrome [41]. Moreover, Folkesson *et al.* demonstrated that in three ALI models induced by HCl instillation, LPS instillation 16 h, or live Escherichia coli instillation, the A_2A_R agonist GW328267C significantly decreased pulmonary edema formation and restored alveolar fluid clearance [42]. These data, coupled with the observation that A_2A_R KO mice had a decrease in overall lung function, which manifested as a decrease in arterial blood oxygen tension, established A_2A_R as a critical factor in limiting inflammatory lung injury and acute lung failure [17]. Consistent with these reports, we confirmed that intratracheal injection of LPS into A_2A_R KO mice led to a markedly increased inflammatory response when compared with WT mice. More importantly, we found the anti-inflammatory protective effect of SIN was eliminated in A_2A_R KO mice. Combination with increasing A_2A_R expression by SIN in WT mice, these results suggest that SIN played its role in ALI in an A_2A_R-dependent manner. Some researches have indicated that A_2A_R gene expression could be regulated by several factors including transcriptional factor [43], microRNA [44], and also A_2A_R agonist [42]. However, in this study, we have not elucidated whether SIN directly stimulated A_2A_R expression as a potential agonist, or indirectly upregulated A_2A_R expression via modulating some transcriptional factors or microRNAs. Accordingly, further investigation should be done for this issue.

Because the infiltration of neutrophils associated with inflammatory cytokine release is a hallmark event in the progression of ALI [45], we sought to determine the mechanism for the A_2A_R-associated SIN effect in LPS-stimulated murine neutrophils. It is well known that A_2A_R is a Gs protein-coupled receptor. Its activation results in an increase in intracellular cAMP levels, which triggers PKA activation to phosphorylate cAMP responsive element-binding protein (CREB). Phosphorylation/activation of CREB has been shown to compete with nuclear factor-kB (NF-κB) p65 for an important co-factor, CBP [46]. Therefore, phosphorylated CREB was proposed to mediate the anti-inflammatory effect of the A_2A_ receptor [47], [48], and inhibition of NF-κB by A_2A_ receptor activation during acute inflammation *in vivo* was demonstrated [49]. CREB may also inhibit the transcriptional activity of NF-κB and subsequently suppress cytokine expression (e.g., tumor necrosis factor) in immune cells [47]. In the present study, we demonstrated that the upregulation of A_2A_R by SIN was accompanied with an increase in cAMP. Similar to the effect of the A_2A_R gene knockout, the PKA inhibitor H-89 could also block the anti-inflammatory effect of SIN in LPS-stimulated neutrophils. These data suggest that A_2A_R-cAMP-PKA signaling, which is reported to suppress NF-κB activity as described above, is involved in the anti-inflammatory role of SIN. This finding is supported by the reports from Wang *et al.* [50], which suggested that the inhibition of NF-κB activity mediates the effects of SIN on cytokine expression in macrophages.

Taken together, our study demonstrates a protective role of SIN in ALI. Upregulation of A_2A_R expression and provoking the downstream cAMP-PKA signal pathway are found to be involved in this protection from SIN in ALI.

## Conclusion

In summary, the present study demonstrates three novel points: (1) SIN attenuates inflammation and lung damage in ALI; (2) SIN upregulates A_2A_R expression in lung tissues in ALI; and (3) A_2A_R-cAMP-PKA signaling is involved in the anti-inflammatory and protective effects of SIN in ALI. These findings elaborate a novel mechanism for SIN in disease and also provide a potential strategy for the clinical treatment of ALI.
